# Medical News

**Published:** 1854-09

**Authors:** 


### THE MEDICAL EXAMINER.

PHILADELPHIA, SEPTEMBER, 1854.

### MEDICAL NEWS.

During the ten weeks previous to the one ending August 26th, in which cholera has prevailed in our city, 453 deaths have been registered from that disease. Total mortality, 3746. The last weekly return exhibited a mortality of only 22 from cholera,—a considerable decrease upon the week previous. Considering the hot and sultry summer we have just passed through, and the very general neglect of sanitary measures, both public and private, we have great cause for gratitude in having escaped a violent visitation of this epidemic.

---

The following remarks upon Endocarditis are from a review by Prof. Lebert, of MM. Rilliet et Barthez' Treatise on the Diseases of Children, contained in the Gazette Medicale de Paris, No. 18. Were not the authority from which these statements issue so almost unimpeachable, we should pass them by as merely idle remarks; as it is, we give them te our readers for what they are worth.

"We have attentively perused everything which these authors say on acute endocarditis; but we must declare that we have not found, either in the anatomical description or in the symptomatology, the proof which they have observed of true endocarditis, a disease which we regard as very rare, especially if we omit fibrinous deposits of membranous form on the valves and endocardium, as well as all incidental stains. We have never yet been able to see a true endocarditis. We have not even seen it produced by incising the endocardium with instruments introduced into the right ventricle through the jugular vein. Once, in fact, our instrument, ending in a watchspring, broke, and the spring remained buried in the substance of the heart during nearly twelve days. The dog on which we were experimenting continued in good health; he had no 'bruit de souffle,' and on killing him, we found the endocardium healthy. A few coagula only adhered to the surface of the spring. We are convinced that all the doctrines upon endocarditis must be reviewed."

---

Dr. J. Lawrence Smith has been appointed Professor of Chemistry in the Medical Department of the University of Louisville, *vice* Prof. Silliman resigned.

Prof. Samuel G. Armor succeeds Prof. Lawson in the chair of Practice of Medicine in the Ohio Medical College. Dr. John A. Warder succeeds Dr. Locke in the chair of Chemistry in the same Institution.

Dr. Harrison, of St. Louis, has been appointed Professor of Materia Medica in the Cincinnati Medical College, a new institution.

Dr. Joseph Parrish, brother of the late Dr. Isaac Parrish, has been appointed to the chair of Obstetrics in the Philadelphia College of Medicine. Dr. Parrish is a physician of much ability and experience, and is favorably known throughout the country as editor of that excellent journal, The New Jersey Medical Reporter. We understand that the present faculty of this Institution have purchased the entire corporate powers and privileges of the old proprietors.

Dr. John P. Gray has been appointed Superintendent of the State Lunatic Asylum, at Utica, in the place of Dr. N. D. Benedict, resigned.

---

Compliment to Dr. N. D. Benedict.—At the Annual Meeting of the Oneida County Medical Society, held July 11, 1854, resolutions were passed highly complimentary to Dr. Nathan D. Benedict, late Superintendent of the New York State Lunatic Asylum, expressing great regret that circumstances should have influenced him to resign a place, for the responsible and arduous duties of which he was so pre-eminently qualified, and which he discharged with so much credit to himself; and conveying the best wishes of the Society for his continued health, prosperity, and usefulness.

